# Reoperation following vitrectomy for diabetic vitreous hemorrhage with versus without preoperative intravitreal bevacizumab

**DOI:** 10.1186/s12886-019-1179-x

**Published:** 2019-09-13

**Authors:** Xuting Hu, Qintuo Pan, Jingwei Zheng, Zongming Song, Zongduan Zhang

**Affiliations:** 1 Eye Hospital, Wenzhou Medical University, 270 Xueyuan Road, Wenzhou, Zhejiang 325027 People’s Republic of China; 2 Henan Eye Institute, Henan Eye Hospital, Henan Provincial People’s Hospital and People’s Hospital of Zhengzhou University, 7 Weiwu Road, Zhengzhou, Henan 450003 People’s Republic of China

**Keywords:** Vitrectomy, Intravitreal bevacizumab, Proliferative diabetic retinopathy, Vitreous hemorrhage, Reoperation

## Abstract

**Background:**

To compare the reoperation rate in patients with vitreous hemorrhage (VH) secondary to proliferative diabetic retinopathy (PDR) with or without preoperative intravitreal bevacizumab (IVB).

**Methods:**

In this retrospective study, 280 patients (362 eyes) with diabetic VH were divided into a group that received preoperative IVB and a group that did not receive preoperative IVB. According to B-scan or color Doppler ultrasonography, the eyes were grouped as a VH group and a tractional retinal detachment (TRD) group. The reoperation rate, visual and anatomical outcomes of treatment were evaluated after 6 months.

**Results:**

There were 17.4% of eyes in the VH group that did not receive preoperative IVB later required additional vitrectomy, while only 7.7% of the eyes in the VH group that received preoperative IVB required additional vitrectomy (*P* = 0.025). There were 45.5% of eyes in the TRD group that did not receive preoperative IVB had no reoperation, while only 21.4% of the eyes in the TRD group that received preoperative IVB had no reoperation (*P* = 0.004). The patients with one operation achieved better vision than those required reoperations in the VH group (*P* = 0.038) and TRD group (*P* = 0.019).

**Conclusions:**

Preoperative IVB significantly reduced the re-vitrectomy rate in patients with VH without TRD, but there was an increase in the reoperation rate in patients with VH combined with TRD.

## Background

Diabetic vitreous hemorrhage (VH) is a common complication of severe proliferative diabetic retinopathy (PDR) and can cause significant vision loss [1]. Historically, treatment for PDR has included panretinal photocoagulation (PRP), cryopexy, and pars plana vitrectomy (PPV) [1–3]. The individualized treatment approach depends on VH status and the patient’s visual acuity [4]. In 2006, Spaide and Fisher reported rapid VH clear-up was obtained after intravitreal bevacizumab (IVB) in two cases of PDR [5]. Since then, intravitreal injection of anti-vascular endothelial growth factor (anti-VEGF) agents have been used widely in the treatment of different manifestations of PDR [3, 4, 6–9]. Anti-VEGF therapy can help the neovascular vessels regress preventing further bleeding, facilitate the absorption of vitreous hemorrhage for application of PRP and may reduce the need for vitrectomy [3, 10]. Vitrectomy is also considered to be deferred temporarily in patients with previous PRP or complete posterior vitreous detachment. However, a patient with nonabsorbent VH is usually a candidate for vitrectomy early, especially with tractional retinal detachments (TRD) and with no history of PRP treatment [4].

The use of IVB as an adjunct before vitrectomy has been routinely recommended over the last 10 years. Numerous reports have revealed that preoperative IVB could reduce intra-operative bleeding, iatrogenic retinal breaks, duration of surgery, early postoperative VH, and neovascular glaucoma (NVG) [11–16]. But Arevalo and colleagues reported that TRD occurred in 11/211 (5.2%) eyes with PDR pretreated with IVB more than a week before the surgery; this suggested an increased risk of TRD with this medication. A potential disadvantage of preoperative anti-VEGF therapy can be the progression of fibrosis and membrane contraction [17]. The beneficial and adverse effects of IVB as a preoperative adjunct in the patients with dense diabetic VH who are candidates for vitrectomy are not thoroughly discussed.

At present, a small gauge transconjunctival sutureless vitrectomy system is considered to reduce postoperative complications after diabetic vitrectomy because of the smaller scleral incision, smaller yet more elegantly designed and sturdy surgical instruments, efficiency of peripheral vitrectomy, and less frequency of postoperative hypotony [18]. However, even with improvements in surgical techniques and the use of preoperative anti-VEGF drugs, a significant number of patients require one or more vitrectomy procedures for postoperative diabetic VH, retinal detachment, or NVG [18–20]. The purpose of this study was to evaluate the reoperation rate in patients with VH or TRD in patients that received preoperative IVB versus those who did not receive preoperative IVB.

## Methods

Consecutive patients aged ≥18 years with either type 1 or type 2 diabetes mellitus who underwent primary vitrectomy for recently developed diabetic VH with or without TRD were included. The operations and examinations were performed at the Eye Hospital of Wenzhou Medical University between January 2010 and June 2017. The routine use of preoperative intravitreal bevacizumab for the diabetic VH in our hospital began in early 2014. We reviewed all electronic medical records of these patients and included the eyes if vitrectomy was performed for nonabsorbent VH with or without preoperative IVB. The nonabsorbent VH lasted for at least 4 weeks and showed no signs of clearance. The IVB + PPV group was drawn from consecutive patients undergoing preoperative IVB plus PPV for diabetic VH from 2014 to 2016, and the PPV alone group was chosen from 2010 to 2013. The exclusion criteria were a previous history of vitrectomy, VH for other reasons, history of diabetic macular edema, macular hole, retinal ischemia found during surgery, detachment with macula-off for more than 6 months, significant corneal opacity and follow-up less than 6 months. The study followed the principles of the Declaration of Helsinki and was approved by the institutional review board and ethics committee of Wenzhou Medical University.

The study subjects included 362 eyes from 280 patients who had undergone PPV for diabetic VH. The macula-involving or macula-threatening TRDs were confirmed when preretinal membranes were observed to exert traction on macular or paramacular areas on B-scan or color Doppler ultrasonography preoperatively. All patients underwent complete ophthalmic examinations, including refraction and best-corrected visual acuity (BCVA) measurements, slit-lamp exams conducted with a 90-D lens, intraocular pressure (IOP) by noncontact tonometry (CT-90A, Topcon, Japan), ultra-widefield color fundus photography (Optos, Dunfermline, United Kingdom), and optical coherence tomography (OCT) (Spectralis HRA OCT; Heidelberg Engineering, Heidelberg, Germany) before surgery and at one, three, and 6 months after surgery, as well as in final follow-ups. The patients with preoperative IVB received intravitreal bevacizumab (1.25 mg, 0.05 ml) in the superior temporal quadrant 4 mm behind the limbus with a sterile syringe before surgery. The vitrectomy was executed 4 days to 21 days (mean 7.2 days) after injection in the IVB + PPV group. All patients underwent standard 23-gauge 3-port PPV, which were performed by one experienced surgeon (Z.M. song). After core vitrectomy without using triamcinolone acetonide, peripheral vitrectomy and vitreous base shaving were performed under a wide-angle viewing system and scleral depression. Fibrovascular membranes and any traction were dissected cautiously with a 23G high-speed vitrectomy cutter instead of vitreoretinal scissors and forceps. Additional procedures such as endodiathermy and laser photocoagulation were performed if needed. In phakic patients aged ≥50 years, phacoemulsification and intracapsular acrylic foldable intraocular lens implantation were performed.

One hundred and nine eyes without TRD did not receive IVB before vitrectomy and were grouped as ‘VH without IVB’ or Group 1. One hundred and seventeen without TRD received IVB before vitrectomy and were grouped as ‘VH with IVB’ or Group 2. Sixty-six eyes with TRD did not receive IVB before vitrectomy and were grouped as ‘TRD without IVB’ or Group 3. Seventy eyes with TRD received IVB before vitrectomy and were grouped as ‘TRD with IVB’ or Group 4.

In this study, the reoperations after diabetic vitrectomy included silicone oil removal, repeated IVB, repeated vitrectomy, and anti-neovascular glaucoma surgery. In China, anti-VEGF treatment needs to be performed in the operating room, and the cost is not covered by medical insurance, which incurs additional costs. Therefore, repeated IVB was recorded as a reoperation in this study. Meanwhile, the four patients who underwent cataract surgery alone after the primary vitrectomy were excluded. Visual acuity was measured 6 months after the final vitrectomy or removal of silicone oil using the standard logarithmic vision chart and was analyzed on a logarithm of the minimum angle of resolution (LogMAR) scale, where counting fingers (CF), hand motion (HM), light perception (LP), and no light perception (NLP) were assigned values of 2.1, 2.4, 2.7, and 3.0, respectively [21].

Statistical analysis was performed with SPSS software version 19.0 (SPSS, Inc., Chicago, IL, USA). Quantitative data were presented as a mean ± standard deviation, whereas qualitative variables were expressed using percentages. For statistical analysis, the generalized estimating equation (GEE) is used to explain the possible correlation between the left and right eyes within the subject. Baseline characteristics and reoperation rates were compared with a GEE approach. Kaplan-Meier survival analysis was used to calculate the cumulative incidence of reoperation after diabetic vitrectomy. Kruskal-Wallis tests were used to compare preoperative and postoperative BCVA and central retinal thickness. Categorical variables were studied using the Fisher’s exact test or Chi-squared test (depending on the frequencies). A *p* < 0.05 was considered to be statistically significant.

## Results

**Fig. 1:**
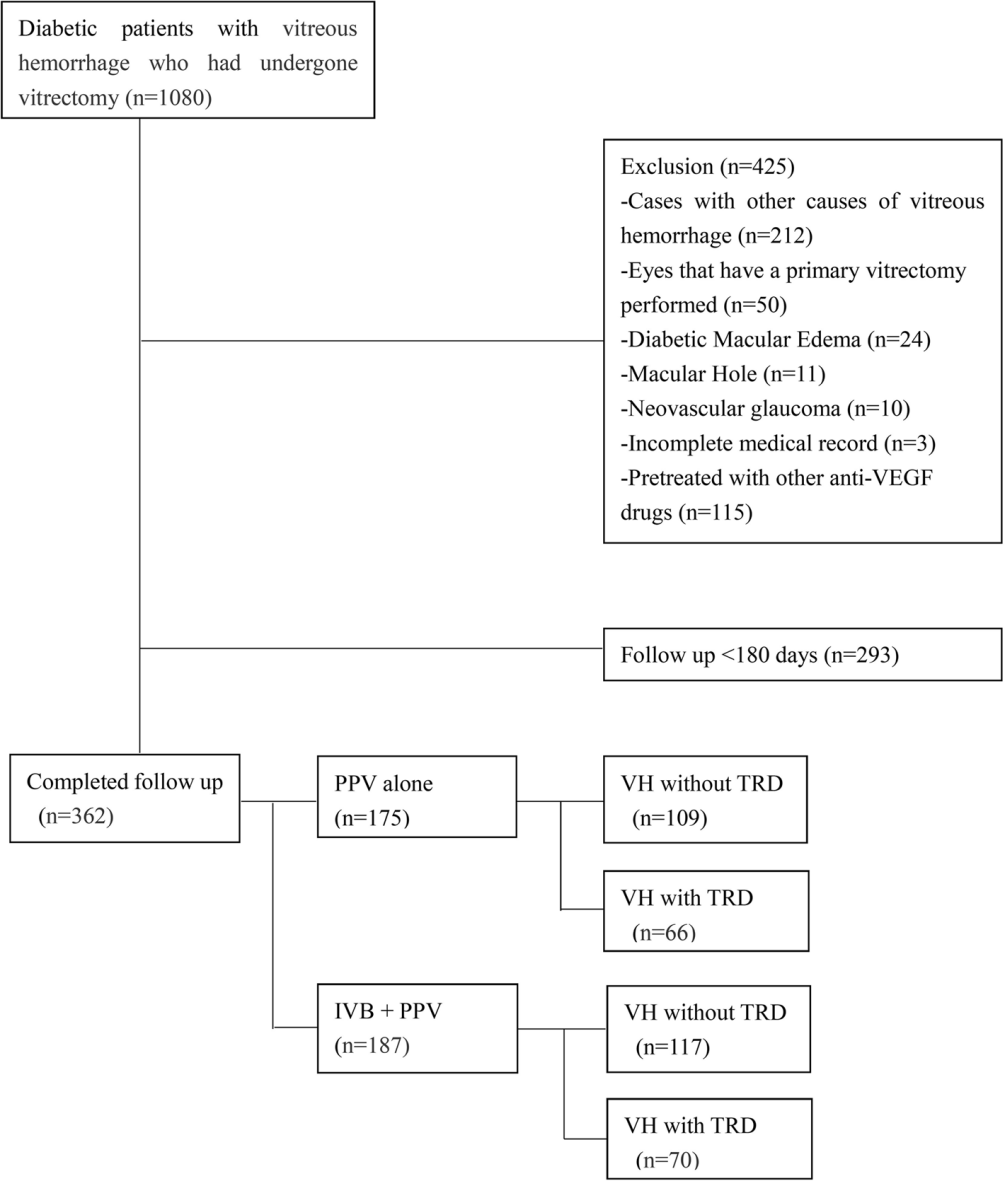
Flow chart of enrolled patients. Anti-VEGF = anti-vascular endothelial growth factor, PPV = pars plana vitrectomy, IVB = intravitreal bevacizumab, VH = vitreous hemorrhage, TRD = tractional retinal detachments

**Fig. 2:**
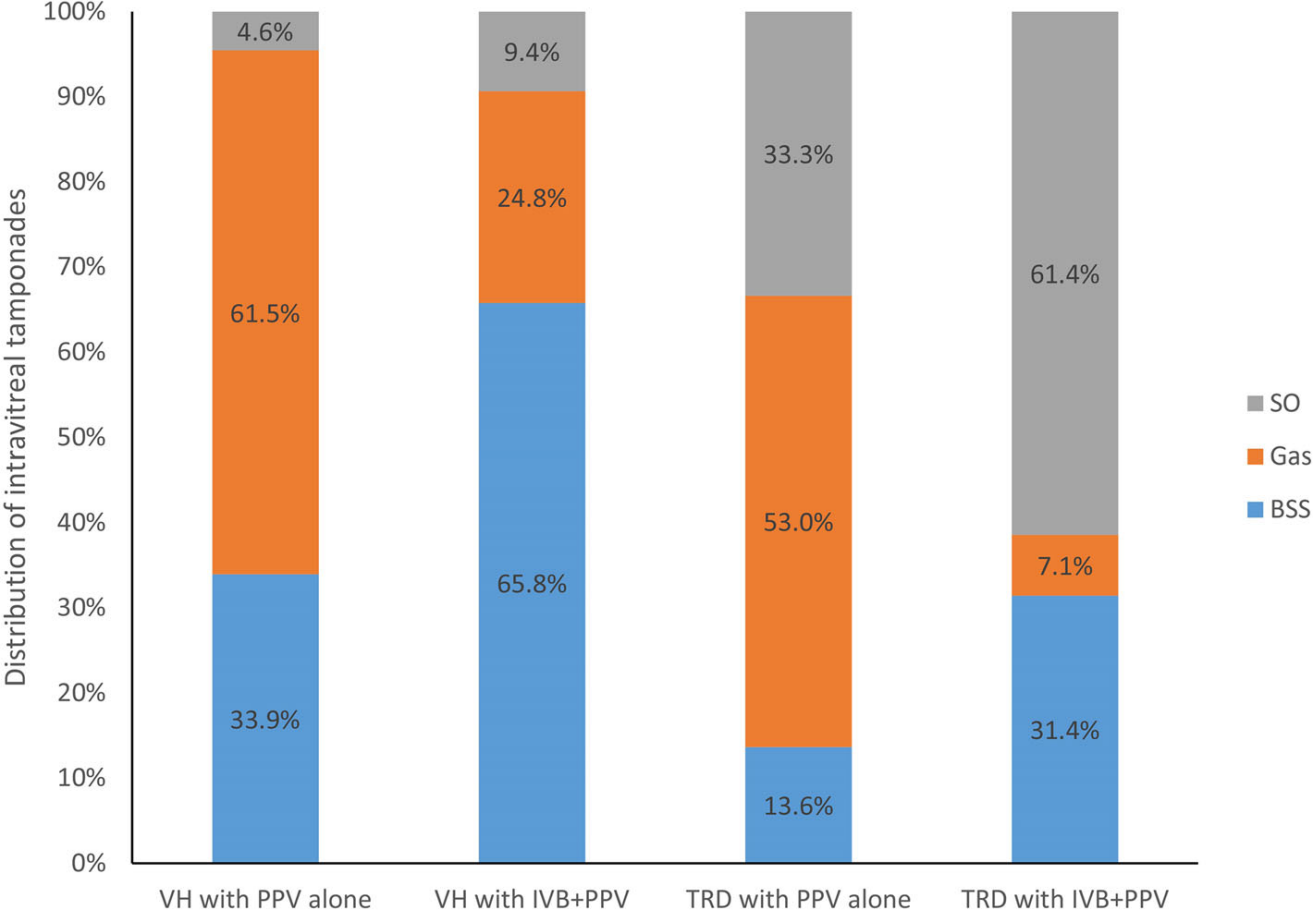
Bar graph showing the use of endotamponades at the end of surgery. SO = silicone oil, Gas = air or perfluoropropane, BSS = balanced salt solution, PPV = pars plana vitrectomy, IVB = intravitreal bevacizumab, VH = vitreous hemorrhage, TRD = tractional retinal detachments

We reviewed 1080 patients with VH who had underwent vitrectomy, of whom we obtained 362 eyes of 280 patients for this review (Fig. 1). Individuals who were lost to follow-up were generally older and had a smaller percentage of TRD compared with those who were not lost to follow-up (Table 1). There were 152 female and 128 male patients. Mean age was 54.8 years (range 25–84 years). Preoperative IVB was performed in 187 eyes, and 175 eyes underwent diabetic vitrectomy alone. Preoperative characteristics of the patients are summarized in Table 2. The use of endotamponades at the end of surgery is shown in Fig. 2.

**Table 1:** Characteristics of eyes with complete and incomplete follow-up

	Incomplete Follow-up	Complete Follow-up	*P*
Number of eyes	293	362	
Sex, *n* (%)			
Male	140 (47.8)	161 (44.5)	0.505
Female	153 (52.2)	201 (55.5)	
Age, mean (SD), years	56.9 (9.7)	54.8 (10.6)	0.019
Diabetes type, *n* (%)			
Type 1	7 (2.4)	13 (3.6)	0.385
Type 2	286 (97.6)	349 (96.4)	
Duration of DM, mean (SD), years	10.6 (6.8)	11.5 (7.0)	0.263
Duration of symptoms, mean (SD), months	4.3 (4.2)	3.9 (4.2)	0.330
Time from the diagnosis, mean (SD), months	2.7 (6.4)	3.1 (7.5)	0.462
HbA_1c_, mean (SD), %	7.7 (1.7)	8.0 (1.8)	0.153
Body mass index, mean (SD), (kg/m^2^)	23.99 (3.10)	23.54 (2.99)	0.181
History of previous PRP, *n* (%)	97 (49.5)	98 (27.1)	0.233
Pseudophakia, *n* (%)	24 (8.2)	24 (6.6)	0.464
Tractional retinal detachments, *n* (%)	77 (26.3)	136 (37.6)	0.004
Preoperative visual acuity, mean (SD), logMAR	1.72 (0.71)	1.71 (0.69)	0.896
Follow-up, mean (SD), months	2.3 (1.8)	29.1 (16.6)	< 0.001

*SD* standard deviation, *DM* diabetes mellitus, *HbA1c* hemoglobin A1c, *PRP* panretinal photocoagulation, *logMAR* logarithm of the minimum angle of resolution

**Table 2:** Baseline Characteristics of Patients with Complete Follow-up

	PPV alone Group	IVB + PPV Group	*P*
Number of eyes	175	187	
Sex, *n* (%)			
Male	74 (42.3)	87 (46.5)	0.564
Female	101 (57.7)	100 (53.5)	
Age, mean (SD), years	55.7 (10.8)	53.8 (10.4)	0.083
Diabetes type, *n* (%)			
Type 1	8 (4.6)	5 (2.7)	0.402
Type 2	167 (95.4)	182 (97.3)	
Duration of DM, mean (SD), years	11.4 (7.0)	11.5 (7.1)	0.823
Duration of symptoms, mean (SD), months	3.8 (4.4)	4.0 (3.9)	0.531
Time from the diagnosis, mean (SD), months	3.2 (7.7)	3.0 (7.2)	0.690
HbA_1c_, mean (SD), %	8.2 (2.0)	7.9 (1.6)	0.342
Body mass index, mean (SD), (kg/m^2^)	23.6 (3.1)	23.5 (2.9)	0.999
History of previous PRP, *n* (%)	50 (28.6)	48 (25.7)	0.954
Pseudophakia, *n* (%)	11 (6.3)	13 (7.0)	0.758
Tractional retinal detachments, *n* (%)	66 (37.7)	70 (37.4)	0.908
Preoperative visual acuity, mean (SD), logMAR	1.76 (0.68)	1.67 (0.70)	0.987
Follow-up, mean (SD), months	30.2 (18.8)	28.1 (14.1)	0.103

*PPV* pars plana vitrectomy, *IVB* intravitreal bevacizumab, *SD* standard deviation, *DM* diabetes mellitus, *HbA1c* hemoglobin A1c, *PRP* panretinal photocoagulation, *logMAR* logarithm of the minimum angle of resolution

### Primary outcomes

**Fig. 3:**
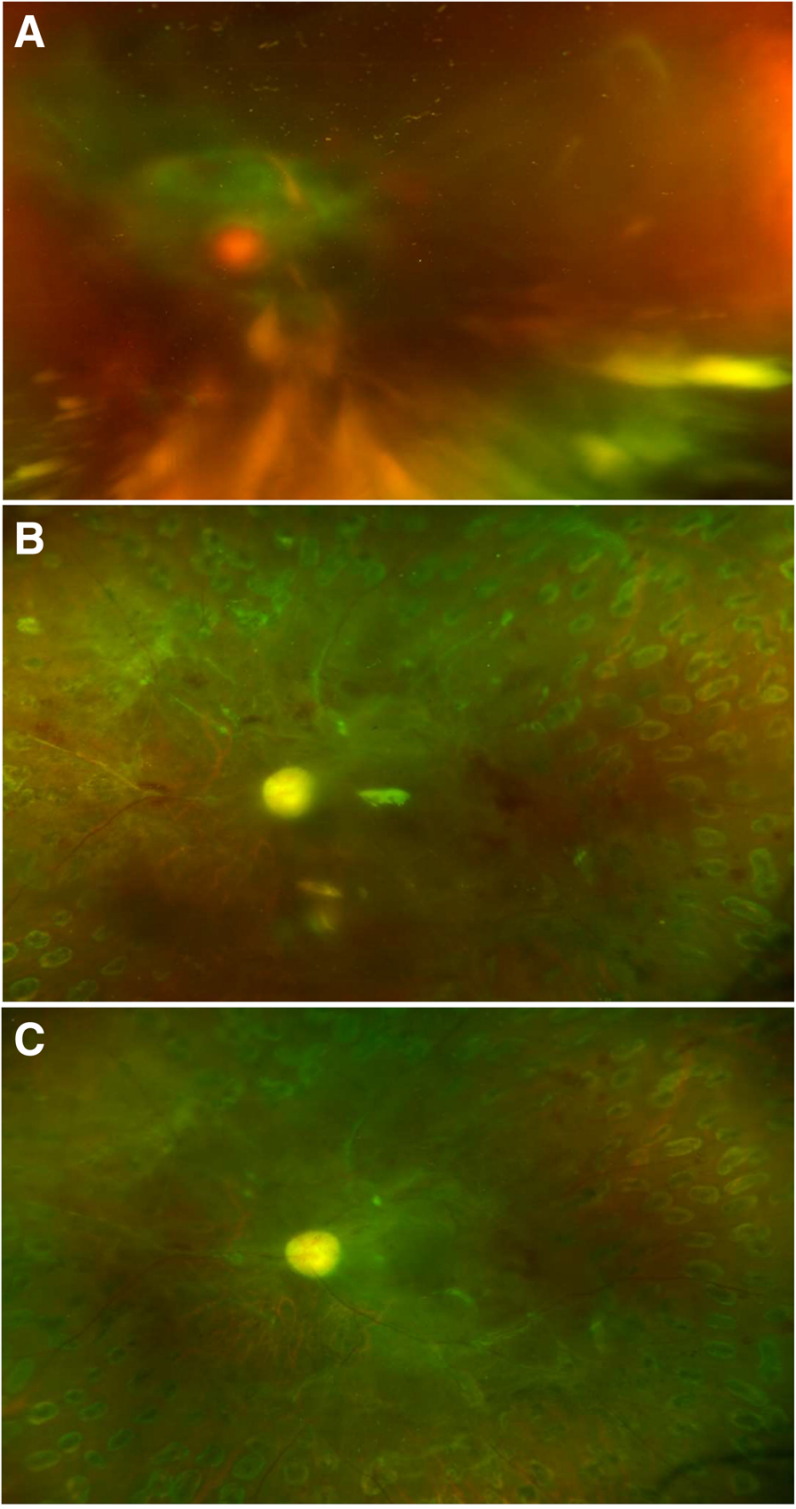
**a** Left eye of a female with type 2 diabetes showing vitreous hemorrhage with tractional retinal detachment and visual acuity of hand motions. **b** She received preoperative intravitreal bevacizumab a week before pars plana vitrectomy with silicone oil tamponade. **c** Three months after the removal of silicone oil, this eye showed visual acuity of finger counting with obvious optic disc pallor

**Fig. 4:**
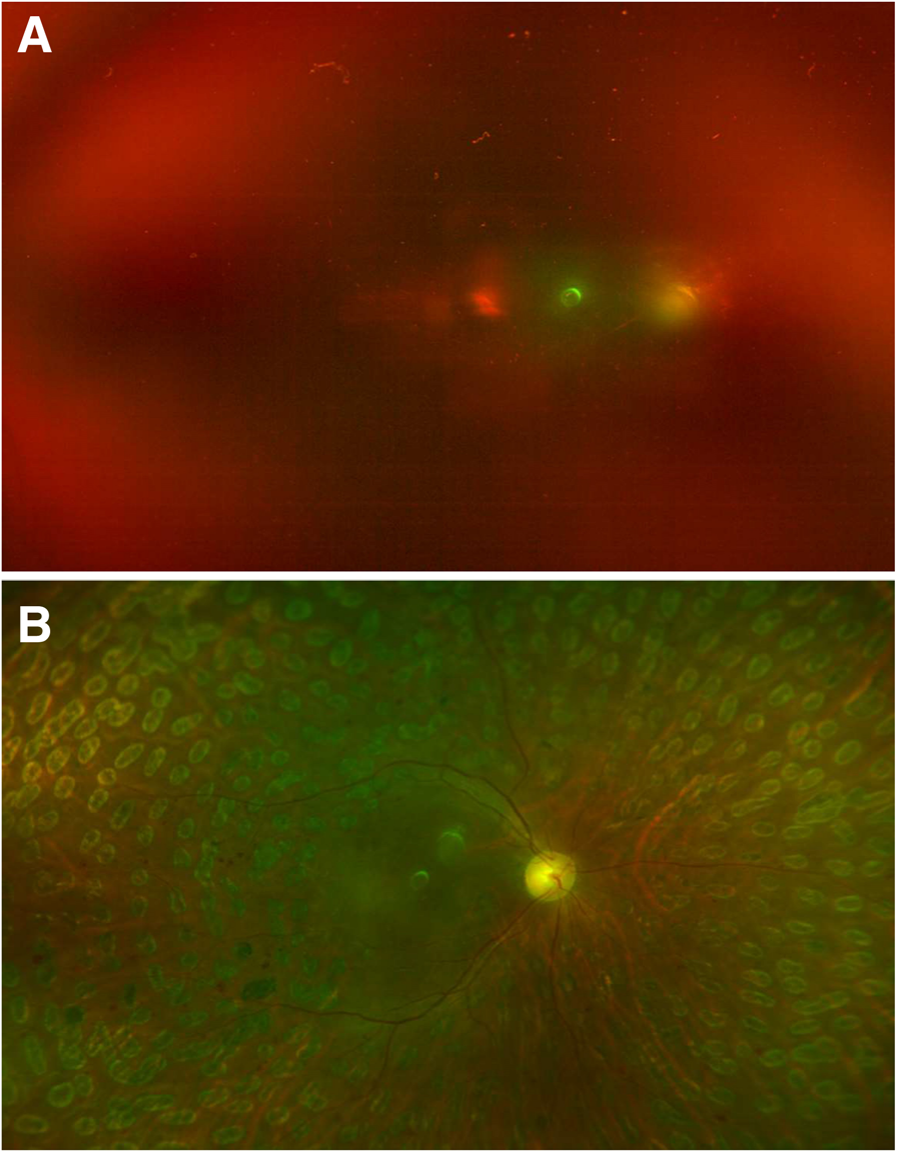
**a** Right eye of a female with type 2 diabetes showing vitreous hemorrhage without tractional retinal detachment and visual acuity of finger counting. **b** She received preoperative intravitreal bevacizumab a week before pars plana vitrectomy with gas tamponade and improved to 20/50 at 6-month follow-up after surgery

**Fig. 5:**
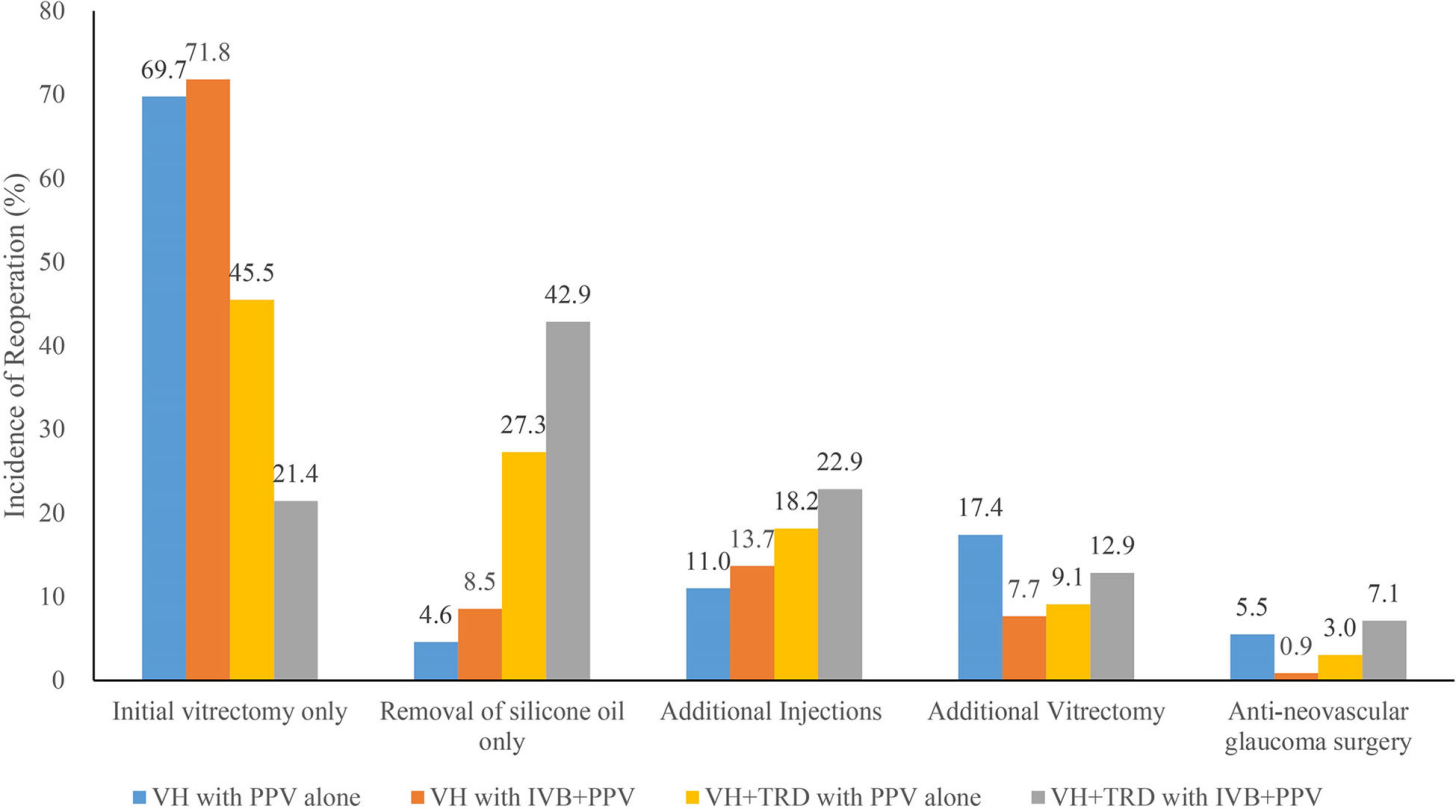
Bar graph showing the final reoperation rate at the last follow up visit. PPV = pars plana vitrectomy, IVB = intravitreal bevacizumab, VH = vitreous hemorrhage, TRD = tractional retinal detachments

The postoperative complications including postoperative VH, elevation of IOP to more than 30 mmHg, retinal detachment, macular edema, epiretinal membrane, NVG and endophthalmitis were recorded in 39.4% (43/109) of the eyes in Group 1, 28.2% (33/117) of the eyes in Group 2, 45.5% (30/66) of the eyes in Group 3 and 61.4% (43/70) of the eyes in Group 4, respectively (*P* < 0.001). In the patients with TRD, there were 45.5% (30/66) of patients without IVB that had no reoperation, while only 21.4% (15/70) of patients with preoperative IVB had no reoperation (IVB + PPV vs. PPV: odds ratio (OR) = 2.864, 95% CI 1.386 to 5.916, *P* = 0.004) (Fig. 3). However, in the patients without TRD, there were 69.7% (76/109) of patients without IVB that had no reoperation, while only 71.8% (84/117) of patients with preoperative IVB had no reoperation (IVB + PPV vs. PPV: OR = 0.894, 95% CI 0.617 to 2.048, *P* = 0.703) (Fig. 4). There were 157 eyes in 134 patients that had a repeated operation, mainly because of the removal of silicone oil. There were 17.4% of eyes in the VH group that did not receive preoperative IVB later required additional vitrectomy, while only 7.7% of the eyes in the VH group that received preoperative IVB required additional vitrectomy (*P* = 0.025). Other reasons include re-vitrectomy and anti-neovascular glaucoma surgery. The reoperations in each group are shown in Fig. 5 and Table 3.

**Table 3:** Associations of Preoperative IVB with Reoperation of Vitrectomy for Diabetic Vitreous Hemorrhage

		Reoperation		Additional Silicone Oil Removal		Additional intravitreal bevacizumab		Additional vitrectomy		Additional anti-glaucoma surgery	
	No. of Eyes	OR (95% CI)	*P* Value	OR (95% CI)	*P* Value	OR (95% CI)	*P* Value	OR (95% CI)	*P* Value	OR (95% CI)	*P* Value
VH without TRD											
PPV alone	117	Ref.	–	Ref.	–	Ref.	–	Ref.	–	Ref.	–
IVB + PPV	109	0.894 (0.490–1.631)	0.714	1.578 (0.557–4.473)	0.391	1.294 (0.562–2.978)	0.544	0.394 (0.174–0.893)	0.026	0.119 (0.015–0.951)	0.045
VH with TRD											
PPV alone	70	Ref.	–	Ref.	–	Ref.	–	Ref.	–	Ref.	–
IVB + PPV	66	2.864 (1.386–5.916)	0.004	2.921 (1.410–6.052)	0.004	1.141 (0.534–2.439)	0.734	1.282 (0.431–3.813)	0.655	2.740 (0.529–14.192)	0.230

Adjusted for age and sex. *IVB* intravitreal bevacizumab, *OR* odds ratio, *CI* confidence interval, *VH* vitreous hemorrhage, *TRD* tractional retinal detachment, *PPV* pars plana vitrectomy, Ref. reference group

The primary reason for additional IVB included postoperative supplementary injection in PPV alone group (5.4%), postoperative VH (39.3%), macular edema (42.9%), and NVG (10.7%). One eye was treated with IVB before the second operation for VH combined with TRD. The average time for additional IVB averaged 6.5 months, with a range of 2 days to 32.2 months. This group included seven patients with the postoperative VH who eventually underwent additional vitrectomy even if they underwent reinjection IVB.

**Fig. 6:**
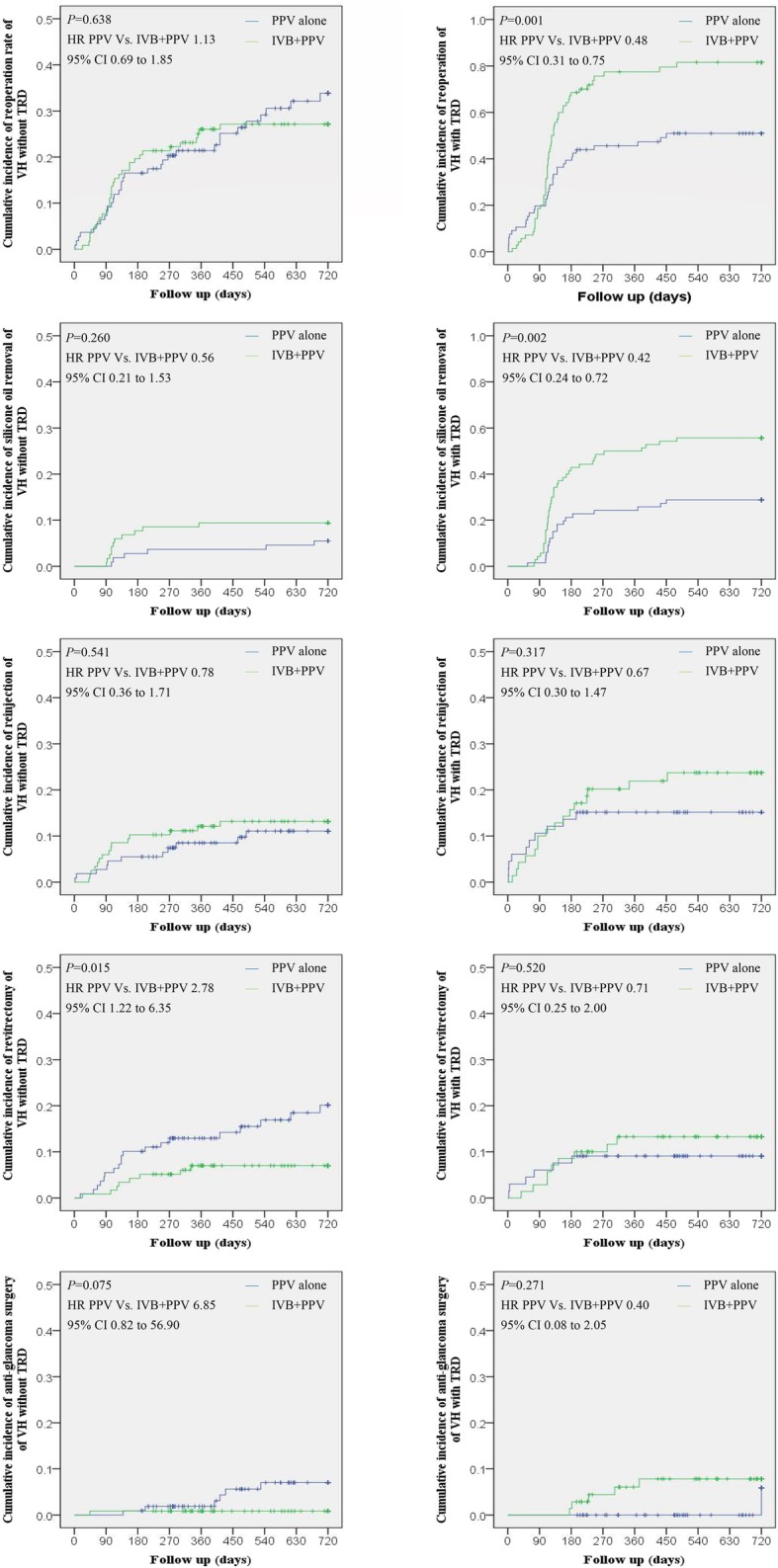
Kaplan-Meier curve for the accumulative incidence of reoperation rate of VH with or without TRD over time. PPV = pars plana vitrectomy, IVB = intravitreal bevacizumab, VH = vitreous hemorrhage, TRD = tractional retinal detachments, HR = hazard ratio, CI = confidence interval

The unplanned PPVs were recorded in 17.4% (19/109) of the eyes in Group 1, 7.7% (9/117) of the eyes in Group 2, respectively (*P* = 0.026), meanwhile, which were recorded in 9.1% (6/66) of the eyes in Group 3 and 12.9% (9/70) of the eyes in Group 4, respectively (*P* = 0.483). The primary reason for additional vitrectomy included postoperative VH in 30 eyes, retinal detachment in 10 eyes, epiretinal membrane in 2 eyes, and endophthalmitis in 1 eye. The time to the second operation averaged 6.4 months, with a range of 2 days to 55 months. Kaplan Meier curves estimated significant differences in the repeated vitrectomy in the patients without TRD (*P* = 0.015) (Fig. 6).

### Secondary outcomes

From baseline to the last follow-up, there were significant improvements in BCVA of − 1.00 LogMAR ±0.58 LogMAR in Group 1 (*P* < 0.001), − 0.88 LogMAR ±0.86 LogMAR in Group 2 (*P* < 0.001), − 0.68 LogMAR ±0.77 LogMAR in Group 3 (*P* < 0.001) and − 0.82 LogMAR ±0.88 LogMAR in Group 4 (*P* < 0.001), respectively. But in the patients with VH groups, patients with one operation achieved better vision (0.66 LogMAR ±0.58 LogMAR) than patients who required reoperation (0.90 LogMAR ±0.76 LogMAR) (*P* = 0.038). Similarly, in the patients with TRD groups, patients without reoperation had better vision (0.79 LogMAR ±0.62 LogMAR) than patients who required reoperation (1.18 LogMAR ±0.86 LogMAR) (*P* = 0.019).

The elevation of IOP to more than 30 mmHg was noted in 30.3% (33/109) of the eyes in Group 1, 10.3% (12/117) of the eyes in Group 2, 30.3% (20/66) of the eyes in Group 3 and 38.6% (27/70) of the eyes in Group 4, respectively (*P* < 0.001). The mean central retinal thickness on OCT at last follow-up was 238 ± 98 μm in Group 1, 230 ± 72 μm in Group 2, 215 ± 120 μm in Group 3 and 251 ± 109 μm in Group 4, respectively (*P* = 0.609).

## Discussion

Vitrectomy has a definite role in the treatment of severe complications of PDR. Over the years, the incidence of postoperative complications of diabetic vitrectomy has been reduced due to significant changes in surgical techniques, instrumentation, experience, and adjunctive use of preoperative IVB. Previous studies about postoperative complications of diabetic vitrectomy have focused on postoperative vitreous cavity hemorrhage (POVCH), retinal detachment, and postoperative NVG [14, 16, 21]. Our study included silicone oil removal as a reoperation because it increased patient costs and affected the final prognosis.

Preoperative IVB is helpful for diabetic vitrectomy and may affect the choice of endotamponade [22]. In patients without TRD, the use of balanced salt solution (BSS) as endotamponade at the end of surgery was more frequent in the IVB + PPV group than in the PPV alone group. Conversely, in patients with TRD, the use of silicone oil was more frequent in the IVB + PPV group, which increased reoperation rate. Previous studies have emphasized the development of TRD after intravitreal injections of anti-VEGF drugs, especially in the absence of prior PRP [17, 23], which is relatively common in China and will affect the choice of postoperative endotamponade. We speculate that the more reoperation rate in the TRD group may be due to the fact that PRP is not widely accepted. In addition, there is no timely vitrectomy after anti-VEGF treatment, which could cause tractional retinal tear and increase the difficulty of surgery and the opportunity to use silicone oil.

Prior to the use of preoperative IVB, Brown reported that 41 (8.5%) of the 484 eyes required additional vitrectomy operation, which caused reoperation, including rhegmatogenous retinal detachment in 18 (44%) of the 41 eyes, recurrent VH in 21 eyes (51%), and glaucoma in two eyes (5%) [24]. Manabe et al. reported that reoperation due to recurrent VH within 4 weeks after surgery was significantly lower (*P* = 0.033) in IVB + PPV group (3.1%, 1/32) than in the sham control group (20.6%, 7/34) [25]. Our study shows that the total re-vitrectomy rate is 11.9% (43/362 eyes) in addition to silicone oil removal surgery. The difference between the studies may be due to different stages of the disease, different preoperative IVB injection time and dose, and doctors and patients’ preferences (including an aversion to various treatment options) that often determine the treatment course.

POVCH is the most common postoperative complication, as high as 75% in some studies without preoperative administration of IVB [16]. In our study, 6.1% (22/362) of eyes required IVB again and 8.3% (30/362) of eyes needed re-vitrectomy because of POVCH. Zhang and colleagues’ meta-analysis included a large number of diabetic patients with nonclearing VH, reported improvements in surgical procedures, reduction of intraoperative complications, and reduction of early postoperative incidence when IVB was given preoperatively compared to when it was not [12].

NVG may be the most devastating result in severe PDR patients, and up to 23% of postoperative patients develop NVG before anti-VEGF is applied [1]. Recent studies have shown that the incidence of postoperative neovascular glaucoma is 4.8% in preoperative anti-VEGF diabetic patients [16]. Also of interest, only 14 of the 362 eyes (3.9%) required surgery for an NVG in our study. However, the comparison of these studies with ours is difficult because of improved systemic diabetes mellitus control, ubiquitous laser treatment, and the development of anti-VEGF therapy. The relatively lower incidence of NVG in our study population may be due to the extensive use of PRP during vitrectomy, higher silicone oil tamponade rates, and postoperative supplementation with anti-VEGF therapy. Combined with the literature and our results, preoperative anti-VEGF treatment of diabetic vitreous hemorrhage could reduce the chance of POVCH, lessen the chance of re-vitrectomy, and reduce the incidence of neovascular glaucoma.

It is controversial whether preoperative anti-VEGF therapy contributes to visual improvement. A meta-analysis suggested that use of IVB has no significant effect on average best-corrected visual acuity (BCVA) at 6 months [14]. In our study, the overall postoperative BCVA in the VH group and the TRD group was in line with expectations. Postoperative BCVA was lower in patients who underwent reoperation than in those who underwent initial vitrectomy, and BCVA was even lower in patients who underwent re-vitrectomy. However, interestingly, in the VH group, the visual acuity after additional anti-VEGF treatment was not lower than that in patients without other anti-VEGF therapy, which may because anti-VEGF treatment can promote vessels regress preventing further bleeding and eliminate macular edema, thus improving or stabilizing eyesight.

We are aware of the limitations of this study concerning its retrospective nature, and our analysis has limitations. The essential defect in this study was the relatively incomplete follow-up of patients more than 3 months after diabetic vitrectomy. The main reason for the lack of data is that many patients were followed up by their local ophthalmologists and were usually taken to the hospital only if there was a renewed loss of vision or a need for reoperation. The second limitation is that the sample size is too small to determine the relationship between anti-neovascular glaucoma surgery and preoperative anti-VEGF therapy and to determine the interval between preoperative IVB and diabetic TRD. Third, the initial severity of the pathology such as proliferative membrane and macular edema in the patients with VH cannot be well recognized before surgery, and only traction retinal detachment can be distinguished according to B-scan or color Doppler ultrasonography, which may lead to selection bias. Finally, our data only reflects the experience of a surgeon at a medical center in China, which may not be generalized to other people.

## Conclusions

In summary, our results confirm preoperative IVB was highly likely to reduce the re-vitrectomy rate in patients with VH without TRD, but it increases the reoperation rate in patients with VH combined with TRD. Given the findings and limitations of the study, randomized controlled trials will help clarify this role, identify the most appropriate treatment options, and identify patients who may need the least intervention to help reduce the burden of treatment.

## Data Availability

All the data supporting our findings is contained within the manuscript.

## Abbreviations

anti-VEGF: anti-vascular endothelial growth factor
BCVA: Best-corrected visual acuity
BSS: Balanced salt solution
CF: Counting fingers
GEE: Generalized estimating eq.
HM: Hand motion
IVB: Intravitreal bevacizumab
LogMAR: Logarithm of the minimum angle of resolution
LP: Light perception
NLP: No light perception
NVG: Neovascular glaucoma
OR: Odds ratio
PDR: Proliferative diabetic retinopathy;
POVCH: Postoperative vitreous cavity haemorrhage
PPV: Pars plana vitrectomy
PRP: Panretinal photocoagulation
TRD: Tractional retinal detachments
VH: Vitreous hemorrhage
